# Mobilization permits reliable ELISPOT detection of long-term memory T cells secreting IFN-γ, IL-4, IL-5, and IL-17 without in vitro expansion

**DOI:** 10.1186/2051-1426-1-S1-P99

**Published:** 2013-11-07

**Authors:** Jaya Ghosh, Alyssa Mills, Marie Wunsch, Edith Karacsony, Wenji Zhang, Jodi Hanson, Paul V Lehmann

**Affiliations:** 1 R&D, Cellular Technology Ltd., Shaker Hts., OH, USA

## Introduction

One of the major deficiencies of all current T cell assays (tetramer, ICS, ELISPOT, proliferation) is that they frequently produce false-negative data. This is the case, for example when one attempts to detect the antigen-specific T cells months or years after vaccinations, for example with tetanus toxoid (TT). While the antigen-specific memory T cells are clearly present in the test subjects mediating long-term immunity, in most individuals the antigen either does not induce a detectable recall response in PBMC at all, or borderline results are obtained. When little-to-no response is detected in PBMC when testing for the recall response, the possible interpretation of such data is either that the frequency of the memory T cells has dropped in PBMC below the detection limit (being around 1 in 100,000 for ELISPOT), or that the memory cells are present in a dormant state that does not permit their detection in standard functional assays.

## Methods

We tested an enhanced medium, CTL-Test™ Plus, that we specially developed to induce dormant memory cells. In a systematic study, this enhanced medium was compared to the non-enhanced, serum-free media, CTL-Test™. The recall response to TT was tested in 42 tetanus vaccinated donors measuring IFN-γ, IL-2, IL-4, IL-5, and IL-17 production by CD4 memory cells in ex vivo ELISPOT assays (without in vitro expansion). The cytokines were measured with the respective ImmunoSpot® Test Kits. The ELISPOTs were counted using an ImmunoSpot® S6 Core reader.

## Results

Tetanus toxoid recalls CD4 cells, CEF peptides CD8 cells. Up to 10-fold increased TT-induced spot counts were seen, without raised spot counts in the medium control, with the CTL-Test™ Plus Medium for most of the subjects tested. The signal was enhanced for detection of the Th1 (IFN-γ, IL-2), Th2 (IL-4, IL-5), and the Th17 (IL-17) subsets of CD4 cells. Signal enhancement was of lesser consistency for CD8 cells activated by the CEF peptide pool, or by individual CEF peptides.

## Conclusions

CTL-Test™ Plus Medium leads to specific signal enhancement for TT-specific CD4 cells of all lineages, possibly for long term CD4 memory cells in general. Notably, about half of the PBMC donors who show no or borderline responses in the Test Medium displayed a significant recall response in the Test Plus Medium. The signal enhancement does not affect most CD8 memory cells that had recent exposure to antigen.

